# Right Hemisphere Regions Critical for Expression of Emotion Through Prosody

**DOI:** 10.3389/fneur.2018.00224

**Published:** 2018-04-06

**Authors:** Sona Patel, Kenichi Oishi, Amy Wright, Harry Sutherland-Foggio, Sadhvi Saxena, Shannon M. Sheppard, Argye E. Hillis

**Affiliations:** ^1^Seton Hall University, South Orange, NJ, United States; ^2^Johns Hopkins Medicine, Baltimore, MD, United States

**Keywords:** prosody expression, stroke, right hemisphere, emotion, communication

## Abstract

Impaired expression of emotion through pitch, loudness, rate, and rhythm of speech (affective prosody) is common and disabling after right hemisphere (RH) stroke. These deficits impede all social interactions. Previous studies have identified cortical areas associated with impairments of expression, recognition, or repetition of affective prosody, but have not identified critical white matter tracts. We hypothesized that: (1) differences across patients in specific acoustic features correlate with listener judgment of affective prosody and (2) these differences are associated with infarcts of specific RH gray and white matter regions. To test these hypotheses, 41 acute ischemic RH stroke patients had MRI diffusion weighted imaging and described a picture. Affective prosody of picture descriptions was rated by 21 healthy volunteers. We identified percent damage (lesion load) to each of seven regions of interest previously associated with expression of affective prosody and two control areas that have been associated with recognition but not expression of prosody. We identified acoustic features that correlated with listener ratings of prosody (hereafter “prosody acoustic measures”) with Spearman correlations and linear regression. We then identified demographic variables and brain regions where lesion load independently predicted the lowest quartile of each of the “prosody acoustic measures” using logistic regression. We found that listener ratings of prosody positively correlated with four acoustic measures. Furthermore, the lowest quartile of each of these four “prosody acoustic measures” was predicted by sex, age, lesion volume, and percent damage to the seven regions of interest. Lesion load in pars opercularis, supramarginal gyrus, or associated white matter tracts (and not control regions) predicted lowest quartile of the four “prosody acoustic measures” in logistic regression. Results indicate that listener perception of reduced affective prosody after RH stroke is due to reduction in specific acoustic features caused by infarct in right pars opercularis or supramarginal gyrus, or associated white matter tracts.

## Introduction

A flat tone-of-voice is often interpreted as apathy, displeasure, sadness, or lack of empathy of the speaker, depending on the context. Yet, survivors of right hemisphere (RH) stroke (1–5) and people with certain neurological diseases—e.g., Parkinson’s disease (6–8), frontotemporal dementia (9–13), schizophrenia (14, 15)—may have trouble modulating their tone-of-voice to express emotion, even when they feel joyful or empathetic. Affective prosody (changes in pitch, loudness, rate, and rhythm of speech to convey emotion) communicates the speaker’s emotion and social intent. Thus, impairments in affective prosody can disrupt all daily interactions and interpersonal relationships, as well as influence social behavior (16).

### Neural Regions Supporting Affective Prosody

It has long been recognized that strokes involving the right frontal lobe, particularly posterior inferior frontal cortex, are associated with impaired expression of affective prosody (3, 17). Infarcts in the right temporal lobe are often associated with impaired recognition of affective prosody (3) or impaired recognition and expression (17). Previous studies have identified cortical areas important for expression of emotion through prosody, using either functional MRI (fMRI) of healthy participants (18–22) or lesion-symptom mapping in individuals with focal brain damage (3, 23). Several studies show activation in inferior frontal cortex, specifically during evoked expressions. However, the brain regions involved seem to be dependent on the type of emotion expressed by the speaker (22). Although most studies of affective prosody impairments have focused on cortical regions, one study showed that infarcts that affected the right sagittal stratum (a large bundle of white matter fibers connecting occipital, cingulate, and temporal regions to the thalamus and basal ganglia) interfered with recognition of sarcasm (24). Nevertheless, few studies have identified the role of specific white matter tracts in the neural network underlying emotional expression.

### RH Dorsal and Ventral Stream Regions for Affective Prosody

The majority of studies investigating emotional prosody have focused on the perception rather than the production of emotion in speech. It has been suggested that, similar to the well-established dual-stream model subserving language processing in the left hemisphere (25–27), prosody comprehension proceeds along analogous dual ventral and dorsal streams in the right hemisphere (28). Specifically, it is proposed that the dorsal “how” pathway is critical for evaluating prosodic contours and mapping them to subvocal articulation, while the ventral “what” pathway, which includes the superior temporal sulcus and much of the temporal lobe, maps prosody to communicative meaning. While the research investigating these pathways in affective prosody generation is sparse, it has been proposed that bilateral basal ganglia play an important role in modulation of motor behavior during the preparation of emotional prosody generation, while RH cortical structures are involved in auditory feedback mechanisms during speech production (29).

### Changes in Acoustic Features Associated With Impaired Affective Prosody

Recent advances in acoustic analysis of speech and voice allow characterization of the fundamental frequency (i.e., the high versus low quality of the voice; measures include the mean, range, peak, and variation), intensity (i.e., how loud or soft the voice is; measures include the mean, range, peak, nadir, variation within, and across bandwidths), speech duration (i.e., how fast or slow the speech is), and rhythm (rate, timing, and relative intensity of various speech segments, such as vowels, consonants, pauses, and so on). Any of these features might be affected by focal brain damage, and changes in one or more feature can influence the perception of the emotion or intent of the speaker.

Previous studies have identified changes in acoustic features that are responsible for abnormal affective prosody in Parkinson’s disease (6, 8), schizotypal personality disorder and schizophrenia (15, 30), and frontotemporal dementia (10, 31). Almost all studies report that less pitch variability and slower rate of speech are associated with reduced prosody in these individuals. A reduction in the pitch variability is what is often referred to as “flat affect,” i.e., a “flat” pitch contour or one that is not as variable. Some conditions such as Parkinson’s disease result in a reduced vocal intensity as well, potentially due to an underlying motor control problem (32), resulting in a quieter voice. Taken together, the impact of these changes is a less variable and therefore a more monotone sounding voice. It has yet to be established whether abnormalities in specific acoustic features account for listener perception of impaired affective prosody after RH stroke.

In this study, we hypothesized that: (1) abnormal patterns of specific acoustic features correlate with lower listener rating of emotional expression and (2) these abnormal acoustic features are associated with infarcts of specific RH gray and white matter regions. Since no one-to-one map between each acoustic feature and a specific brain region exists, a standard set of acoustic parameters were investigated based on the features that are known to be affected in pathological conditions, including RH stroke.

## Materials and Methods

### Participants

A consecutive series of 41 acute ischemic RH stroke patients who provided written informed consent for all study procedures were enrolled. Consent forms and procedures were approved by the Johns Hopkins Institutional Review Board. Exclusion criteria included: previous neurological disease involving the brain (including prior stroke), impaired arousal or ongoing sedation, lack of premorbid competency in English, left handedness, <10th grade education, or contraindication for MRI (e.g., implanted ferrous metal). The mean age was 62.7 ± SD 12.5 years. The mean education was 14.4 ± 3.3 years. The mean lesion volume was 37.2 ± 67.0 cc. Participants were 41.5% women. Within 48 h of stroke onset, the participants were each administered a battery of assessments of affective prosody expression and recognition, but in this study we focused on affective prosody expression to test our hypotheses.

### Acoustic Analysis

**Figure 1:**
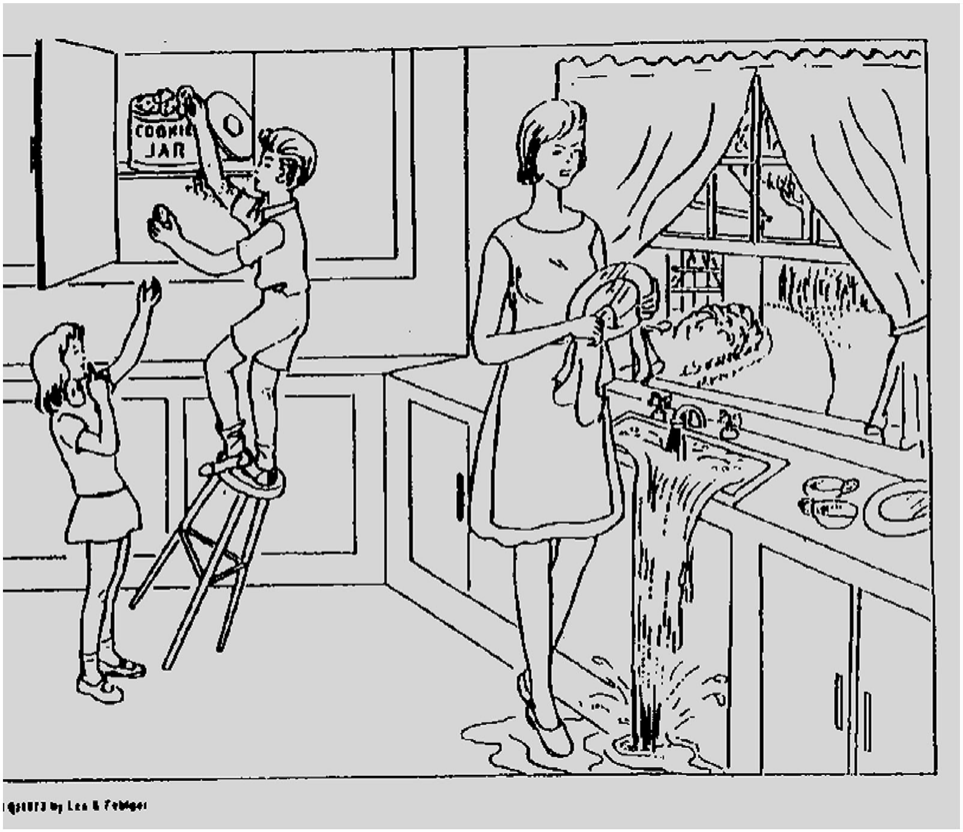
The stimulus for the picture descriptions (the “Cookie Theft” picture).

The speech samples from each participant included a description of the “Cookie Theft” picture, originally from the Boston Diagnostic Aphasia Examination (33). This same picture is also used in the National Institutes of Health Stroke Scale (34, 35). The stimulus is shown in Figure 1. Participants were instructed to describe the picture as if they were telling a story to a child. Participants were prompted to continue (“anything else that you can tell me?”) once. Recordings were made using a head-worn microphone placed two inches from the mouth of the participant. All samples were segmented and converted into mono recordings for analysis in Praat (36). A total of 26 parameters were automatically extracted from the speech samples using customized scripts. The parameters included measurements related to fundamental frequency (*F*_0_), intensity, duration, rate, and voice quality. The full list of parameters is given in Table 1 along with a short description of each measure. Because we had no *a priori* evidence to hypothesize that some of these features would be more affected than others, we included a standard list of features (measures of *F*_0_ and intensity and durations of various parts of speech) as well as a set of features that were either relative to certain frequency bands or parts of speech. We followed the same procedures followed in previous publications; for example, see Ref. (37) for details of the analyses.

**Table 1:** Acoustic measures that were included in the analyses.

Abbreviation	Description
*F*_0_mean	Mean fundamental frequency
*F*_0_sd	Standard deviation of fundamental frequency
*F*_0_max	Max of fundamental frequency
*F*_0_min	Min of fundamental frequency
*F*_0_rg	Range of fundamental frequency
*F*_0_CoV	Coefficient of variation of *F*_0_
INTmean	Mean intensity
INTsd	Standard deviation of intensity
INTmax	Max intensity (95%)
INTmin	Min intensity (5%)
INTrg	Range of intensity
relen1000dB	Relative energy of 1–8 kHz (dB)
relen500dB	Relative energy of 500 Hz to 8 kHz (dB)
alpha_ratio	Relative energy of 1–5 kHz (dB)
H1H2	H1H2 level difference
DurV	Duration of voiced-only parts of speech
DurU	Duration of unvoiced-only parts of speech
DurSil	Duration of silences
Dur	Total duration
DurV/DurS	Duration of voiced segm. over articulated duration
JIT	Jitter
SHIM	Shimmer
hamm	Hammarberg index
mn_int < 1k_VoicedOnly	Mean energy 0–1,000 Hz
HNR	Mean harmonics-to-noise ratio

#### Listener Rating

The emotional expression of the speech samples was rated by 21 healthy volunteers, using a 1–7 scale (from no emotion to very emotional). They were given several practice items with feedback. The mean score for the 21 listeners for each voice sample was used to identify the acoustic features related to the listener ratings of emotional expression.

### Image Analysis

**Figure 2:**
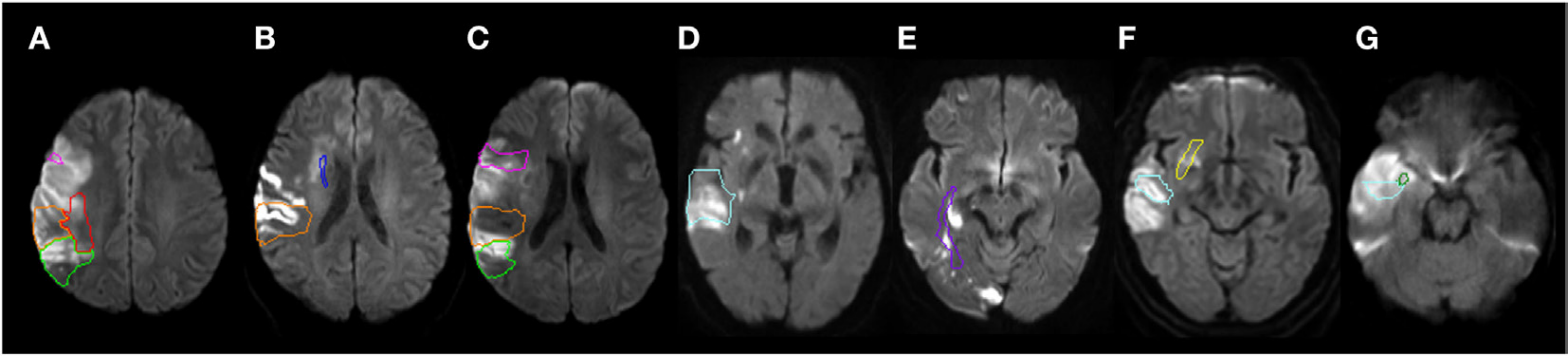
Representative individuals with acute infarction in the selected structures. The structures are color-contoured: inferior frontal gyrus, pars opercularis [pink **(A,C)**], superior temporal gyrus [cyan **(D,F,G)**], supramarginal gyrus [orange **(A,B,C)**], angular gyrus [chartreuse green **(A,C)**], inferior fronto-occipital fasciculus [yellow **(F)**], sagittal stratum [purple **(E)**], superior fronto-occipital fasciculus [blue **(B)**], superior longitudinal fasciculus [red **(A)**], and the uncinate fasciculus [green **(G)**]. Diffusion weighted images were normalized to the JHU-MNI atlas space and pre-defined ROIs were overlaid on the normalized images. Images are all in radiological convention: left side of the figure is the right side of the individual.

Participants were evaluated with MRI diffusion weighted imaging (DWI), fluid attenuated inversion recovery (to rule out old lesions), Susceptibility weighted imaging (to rule out hemorrhage), and T2-weighted imaging to evaluate for other structural lesions. A neurologist (Kenichi Oishi) who was blind to the results of the acoustic analyses identified the percent damage to each of seven gray and white matter regions that have previously been associated with deficits in expression of affective prosody and two control areas that have been associated with deficits in recognition but not expression of prosody (3, 13, 23, 24, 38). The seven regions of interest hypothesized to be related to prosody expression in the RH were: inferior frontal gyrus pars opercularis; supramarginal gyrus; angular gyrus; inferio-frontal-occipital fasciculus, superior frontal occipital fasciculus; superior longitudinal fasciculus (SLF); and uncinate fasciculus. The control areas that have previously been identified as critical for prosody recognition but not production (22, 24, 30) were: superior temporal gyrus and sagittal stratum. The procedure followed previous publications (39–41). In brief, the boundary(s) of acute stroke lesion(s) was defined by a threshold of >30% intensity increase from the unaffected area in the DWI (42, 43) then manually modified to avoid false-positive and false-negative areas by a neurologist (Kenichi Oishi). Kenichi Oishi was blinded to the results of the acoustic analyses to avoid bias in lesion identification. Then, the non-diffusion weighted image (b0) was transformed to the JHU-MNI-b0 atlas using affine transformation, followed by large deformation diffeomorphic metric mapping (LDDMM) (44, 45). The resultant matrices were applied to the stroke lesion for normalization. LDDMM provides optimal normalization to minimize warping regions of interest (37, 38). A customized version of the JHU-MNI Brain Parcelation Map^1^ was then overlaid on the normalized lesion map to determine the percentage volume of the nine regions (Figure 2), using DiffeoMap.^2^

### Statistical Analysis

All analyses were carried out with Stata, version 12 (StataCorp^3^). Acoustic features (from the 26 listed in Table 1) that correlated with mean listener judgments of affective prosody were identified with Spearman correlations. An alpha level of *p* < 0.05, after correction for multiple comparisons (*n* = 26) with Bonferroni correction, was considered significant. Acoustic features that independently contributed to listener rating of emotional expression, after adjustment for other acoustic features and age, were identified with linear regression, separately for men and women speakers, and were used in further analyses as the “prosody acoustic measures.” Then, percent damage to each ROI that independently predicted the lowest quartile of each of the identified prosody acoustic measures were identified using logistic regression. The independent variables included age, sex, education, and percent damage to (lesion load in) each of the nine ROIs (including two control regions). We included age and sex in all multivariable logistic regressions, along with percent damage to each of the five cortical regions of interest and the four white matter bundles of fibers, because age and sex can influence all acoustic features. Because we did not include healthy controls, we defined the lowest quartile of each prosody acoustic measure as abnormal. We chose this definition because we aimed to focus only on the most disrupted prosody for our analyses. Thus, the dependent variables were whether or not a patient’s score on a particular acoustic measure fell in the lowest quartile of the distribution across patients, coded as 0 or 1.

## Results

### Abnormalities in Acoustic Features Associated With Listener Perception of Impaired Affective Prosody

Mean scores (and SDs) for each of the acoustic features for men and women are shown in Table 2. There was no significant difference between men and women in age [male mean = 62.0, female mean = 63.8, *t*(39) = 0.41, ns]. Listener judgments of prosody correlated with certain cues, namely the relative articulation duration, i.e., the relative duration of voiced segments to the total duration of speech segments excluding pauses (Dur_v/s_) (rho = 0.63; *p* < 0.00001) and spectral flatness (SF) (rho = −0.55; *p* = 0.0002). None of the other acoustic features correlated with listener judgment of prosody in univariate analyses.

**Table 2:** Mean and SD for each acoustic measure across sexes.

Acoustic measure	Mean (and SD) for men	Mean (and SD) for women
*F*_0_mean	249.7 (126.3)	227.7 (61.3)
*F*_0_sd	120.2 (37.9)	109.9 (29.5)
*F*_0_max	461.9 (168.9)	475.2 (118.8)
*F*_0_min	110.4 (52.1)	118.3 (37.4)
*F*_0_rg	351.6 (150.5)	357.0 (109.0)
*F*_0_CoV	31.4 (13.3)	23.9 (9.8)
INTmean	65.7 (10.3)	65.7 (4.4)
INTsd	6.4 (3.2)	5.7 (2.6)
INTmax	78.1 (11.7)	81.1 (5.5)
INTmin	46.1 (10.0)	49.3 (7.7)
INTrg	32.0 (8.9)	31.8 (9.0)
relen1000dB	−3.7 (1.0)	−3.2 (1.0)
relen500dB	−7.8 (2.2)	−7.6 (2.2)
alpha_ratio	7.0 (3.0)	9.3 (3.3)
H1H2	−0.60 (4.0)	0.035 (3.7)
DurV	15.0 (11.7)	14.7 (11.6)
DurU	20.2 (16.1)	27.7 (16.4)
DurSil	2.3 (6.3)	2.9 (5.7)
Dur	37.5 (27.4)	45.4 (17.8)
Dur_V/S_	0.43 (0.17)	0.35 (0.20)
JIT	0.046 (0.026)	0.037 (0.015)
SHIM	0.17 (0.038)	0.16 (0.033)
hamm	10.3 (5.9)	14.5 (9.2)
mn_int < 1k_VoicedOnly	64.9 (10.4)	64.9 (4.4)
HNR	6.7 (1.7)	7.2 (1.3)
SF	−3.0 (0.96)	−3.6 (2.5)
Age	62.0 (10.9)	63.6 (14.6)

In multivariable analyses, mean listener rating (from 1 to 7) was best accounted for by a model that included Dur_v/s_, SF, *F*_o_ range, and *F*_0_ coefficient of variation (*F*_0_CoV) of fundamental frequency in both women [*F* (4, 12) = 6.58; *p* = 0.0048; *r*^2^ = 0.69] and men [*F*(5, 18) = 5.13; *p* = 0.0056; *r*^2^ = 0.52]. These identified acoustic measures that correlated with listener judgment of prosody were then considered the “prosody acoustic measures” used in further analyses. The only feature found to be independently associated with rating of emotional expression was Dur_v/s_ (*p* < 0.0001) for women, and SF (*p* = 0.007) for men, after adjusting for other variables (age and the other acoustic features, from the set of 26) (Table 3). Dur_v/s_ was positively correlated with perceived emotional rating in both women (rho = 0.71; *p* = 0.0015) and men (rho = 0.55; *p* = 0.0052), but the correlation was stronger in women. SF was negatively correlated with perceived emotional expression in both women (rho = −0.39; *p* = 0.13) and men (rho = −0.69; *p* = 0.0002), but the association was significant only in men. That is, women (and to a lesser degree, men) who used more voicing were rated as having higher emotional prosody, and men who had higher SF were rated as having lower emotional prosody.

**Table 3:** Results of linear regression to identify “prosody acoustic measures”—measures that contributed to listener rating of affective prosody.

	Coefficient	SE	*t*	*p*-Value	95% CI
**For men**					
*F*_0_CoV	0.0089	0.013	0.67	0.51	−0.04 to 0.02
Dur_v/s_	0.43	1.4	0.30	0.77	−3.4 to 2.6
*F*_0_rg	0.00034	0.0011	0.30	0.77	−0.0028 to 0.0020
SF	−0.74	0.24	−3.04	0.007	−1.3 to −0.23

**For women**					
*F*_0_CoV	0.010	0.022	0.48	0.64	−0.038 to 0.060
Dur_v/s_	6.1	1.3	4.7	<0.0001	3.3 to 8.9
*F*_0_rg	0.0053	0.0030	1.8	0.11	−0.011 to 0.0013
SF	0.20	0.15	1.4	0.20	−0.12 to 0.51

### Lesions and Demographics Associated With Abnormal Acoustic Features

As indicated above, speech samples with the lowest level of each of the four “prosody acoustic measures” were rated as having the lowest affective prosody by healthy listeners. The lowest quartile of SF was predicted by sex, age, lesion volume, and percent damage to the nine RH regions (*X*^2^ = 27; *p* = 0.0081). Sex, lesion volume, damage to inferior frontal gyrus pars opercularis, inferior fronto-occipital (IFO) fasciculus, SLF, and uncinate fasciculus were the only independent predictors, after adjusting for the other variables. The lowest quartile of *F*_0_CoV was predicted by sex, age, lesion volume, and percent damage to the nine RH regions (*X*^2^ = 33; *p* = 0.0005); age and damage to supramarginal gyrus and SLF were the only independent predictors. The lowest quartile of Dur_v/s_ was predicted by sex, age, education, lesion volume, and percent damage to the nine RH regions (*X*^2^ = 25; *p* = 0.02), but none of the variables were independent predictors of Dur_v/s_, after adjustment for other independent variables. The more ventral control regions (STG and sagittal stratum) were not independent predictors of any of the prosody acoustic features in the logistic regression models.

## Discussion

There are two novel and important results of this study. First, we identified abnormal patterns of acoustic features that contribute to diminished emotional expression of RH stroke survivors, as rated by healthy listeners. The features that together best accounted for diminished emotional expression were: the relative duration of the voiced parts of speech (Dur_v/s_), SF, *F*_0_ range, and *F*_0_CoV. The first two features, Dur_v/s_ and SF, are measures of rhythm; the latter two features, *F*_0_ range and *F*_0_CoV, relate to pitch. Several previous studies have shown that *F*_0_ range (31) or *F*_0_ CoV (3, 23, 46) are abnormal in neurological diseases associated with impaired prosody, but most studies have not compared these acoustic features to other acoustic features that might convey emotional expression. We found that Dur_v/s_ was particularly important in emotional expression of female stroke participants, and SF was particularly important in emotional expression of male stroke participants. SF (computed as the ratio of the geometric to the arithmetic mean of the spectral energy distribution) has been shown to be important in conveying happy and sad tone-of-voice (47); see also (48). Differences between sexes might reflect differences in which emotions were rated as less emotional in men versus women. It is possible that men were rated as less emotional mostly on the happy and sad stimuli (which depend on SF), whereas women were rated less emotional on emotions that depend more on less noise or breathiness (captured by Dur_v/s_), such as angry and happy. Our study was not powered to evaluate each emotion separately, so this speculation will need to be evaluated in future research.

The variable of Dur_v/s_ has been less studied than the other prosody acoustic measures we identified, with respect to emotional communication. However, one study showed that vocal fold contact time (which underlies Dur_v/s_) varied substantially between expression of different emotions (49), consistent with a role for the percentage of voiced speech segments in conveying emotion. Yildirim et al. (50) carried out acoustic analysis of transitions from neutral to happy, sad, or angry speech, and found that angry and happy speech are characterized by longer utterance duration, as well as shorter pauses between words, higher *F*_0_, and wider ranges of energy, resulting in exaggerated, or hyperarticulated speech (51); but they did not specifically evaluate Dur_v/s_.

The second important finding is that the measures of acoustic features associated with impaired expression of emotion (“prosody acoustic measures”) were associated with lesion load in right IFG pars opercularis or supramarginal gyrus, or associated white matter tracts, particularly right IFO fasciculus, SLF, and uncinate fasciculus. These findings are consistent with, but add specificity to, the proposal of a dorsal stream for transcoding acoustic information into motor speech modulation for affective prosody expression in the RH and a ventral stream for transcoding acoustic information into emotional meaning for affective prosody recognition (38, 52). The areas we identified that affected prosody acoustic measures, particularly IFG pars opercularis, supramarginal gyrus, and SLF (roughly equivalent to the arcuate fasciculus) are regions often considered to be included in the dorsal stream of speech production in the left hemisphere (53) and the dorsal stream of affective prosody production in the RH (29, 44). Because we focused on affective prosody expression, we did not provide evidence for the role of the proposed ventral stream. However, lesions in relatively ventral areas, including STG and sagittal stratum (white matter tracts connecting basal ganglia and thalamus with temporal and occipital lobes), which served as control regions, were not associated with impaired (lowest quartile) of prosody acoustic measures in multivariable logistic regression. Other studies are needed to evaluate the cortical and white matter regions associated with recognition of affective prosody. One study identified an association between damage to the sagittal stratum and impaired recognition of sarcastic voice (30).

An important role of right inferior frontal gyrus lesions in disrupting affective prosody expression has also been reported by Ross and Monnot (3). Furthermore, in an fMRI study of healthy controls, evoked expressions of anger (compared with neutral expressions) produced activation in the inferior frontal cortex and dorsal basal ganglia (22). Expression of anger was also associated with activation of the amygdala and anterior cingulate cortex (23), areas important for some aspects of emotional processing, such as empathy (31). The role of disruption to specific white matter tract bundles on affective prosody expression has been less studied than the role of cortical regions. One study showed that in left hemisphere stroke patients, deficits in emotional expression that were independent of the aphasic deficit were associated with deep white matter lesions below the supplementary motor area (which disrupt interhemispheric connections through the mid-rostral corpus callosum) (54). Here, we identified RH white matter tracts that are critical for expression of emotion through prosody, including IFO fasciculus, SLF, and uncinate fasciculus. Results indicate that affective prosody production relies on right IFO fasciculus, SLF, uncinate fasciculus, as well as supramarginal gyrus and inferior frontal gyrus pars opercularis.

Limitations of our study include the relatively small number of patients, which also limited the number of regions of interest we could evaluate. The small number of patients also reduces the power to detect associations between behavior and regions that are rarely damaged by stroke. Thus, there may be other areas that are critical for expression of emotion through prosody. We also did not analyze speech of healthy controls for this study, so we defined as “abnormal” those who were rated as having low-emotional expression by healthy controls. Despite its limitations, this study provides new information on specific gray and white matter regions where damage causes impaired expression of emotion through prosody.

## Ethics Statement

This study was carried out in accordance with the recommendations of the Johns Hopkins Institutional Review Board with written informed consent from all subjects. All subjects gave written informed consent in accordance with the Declaration of Helsinki. The protocol was approved by the Johns Hopkins Institutional Review Board. Analysis of the voice data was also performed under a protocol approved by the Seton Hall University Institutional Review Board.

## Author Contributions

SP responsible for acoustic analysis, design of the study, and editing of the paper. KO responsible for image analysis and editing of the paper. AW, HS-F, and SS responsible for data collection and analysis, editing of the paper. AH responsible for data analysis, design of the study, and drafting of the paper.

## Conflict of Interest Statement

The authors declare that the research was conducted in the absence of any commercial or financial relationships that could be construed as a potential conflict of interest.

## Footnotes

^1^http://cmrm.med.jhmi.edu (Accessed: January 6, 2017).

^2^www.MRIstudio.org (Accessed: January 6, 2017).

^3^www.stata.com (Accessed: January 6, 2017).
